# A Pandemic Lesson for Global Lung Diseases: Exacerbations Are Preventable

**DOI:** 10.1164/rccm.202110-2389CI

**Published:** 2022-02-22

**Authors:** William Cookson, Miriam Moffatt, Garth Rapeport, Jennifer Quint

**Affiliations:** National Heart and Lung Institute, Imperial College, London, United Kingdom

**Keywords:** SARS-CoV-2 pandemic, nonpharmaceutical interventions, asthma, COPD, pneumonia

## Abstract

A dramatic global reduction in the incidence of common seasonal respiratory viral infections has resulted from measures to limit the transmission of severe acute respiratory syndrome coronavirus 2 (SARS-CoV-2) during the pandemic. This has been accompanied by falls reaching 50% internationally in the incidence of acute exacerbations of preexisting chronic respiratory diseases that include asthma, chronic obstructive pulmonary disease, and cystic fibrosis. At the same time, the incidence of acute bacterial pneumonia and sepsis has fallen steeply worldwide. Such findings demonstrate the profound impact of common respiratory viruses on the course of these global illnesses. Reduced transmission of common respiratory bacterial pathogens and their interactions with viruses appear also as central factors. This review summarizes pandemic changes in exacerbation rates of asthma, chronic obstructive pulmonary disease, cystic fibrosis, and pneumonia. We draw attention to the substantial body of knowledge about respiratory virus infections in these conditions, and that it has not yet translated into clinical practice. Now that the large scale of benefits that could be gained by managing these pathogens is unmistakable, we suggest that the field merits substantial academic and industrial investment. We consider how pandemic-inspired measures for prevention and treatment of common infections should become a cornerstone for managing respiratory diseases.

Approximately 545 million people worldwide have a chronic respiratory disease, an increase of 40% from 1990 to 2017 (1). The annual costs of healthcare and lost productivity because of chronic obstructive pulmonary disease (COPD) and asthma are €48.4 billion and €33.9 billion, respectively, in the European community; half of this is attributable to exacerbations for both diseases (https://www.erswhitebook.org/). In the United States, the total cost of asthma, including absenteeism and mortality, was $81.9 billion in 2013 (2); 37% of medical costs were attributable to acute episodes. The annual cost of COPD to the U.S. economy was $38.8 billion in 2005 (3). As a consequence, preventing exacerbations of these most common respiratory conditions is of global importance.

## Circulation of Respiratory Viruses during the Pandemic

During the severe acute respiratory syndrome coronavirus 2 (SARS-CoV-2) pandemic, there has been widespread introduction in both the Northern and Southern hemispheres of nonpharmaceutical interventions that included enforced lockdowns, social distancing, border restrictions, school closures, and tracing and isolation of symptomatic individuals (4). Within-season influenza activity has been at historically low prevalence since 2020 (World Health Organization Influenza Update N° 398 [5]), and circulations of human metapneumovirus, enterovirus, adenovirus, respiratory syncytial virus (RSV), and human rhinovirus (HRV) have all been substantially reduced (6). In the United Kingdom, the emergence of SARS-CoV-2 was associated with substantial reductions in the circulation of seasonal respiratory viruses and large differences in the characteristics of viral-associated disease (7).

## Pandemic Effects on Exacerbations

### Asthma

Unexpectedly marked changes in the incidence of acute asthma attacks during the SARS-CoV-2 pandemic have been observed internationally. In the United States, a study of 3,959 children and adolescents with diagnosed asthma found all-cause healthcare encounters decreased significantly during the pandemic compared with the preceding year. This included well-child visits (48.1% during the pandemic vs. 66.6% in the prior year; *P* < 0.01), emergency department visits (9.7% vs. 21.0%; *P* < 0.01), and inpatient admissions (1.6% vs. 2.5%; *P* < 0.01), despite a 100-fold increase in telehealth encounters (8). Asthma exacerbations that required treatment with systemic steroids also decreased (127 vs. 504 exacerbations; *P* < 0.01) (8). A Harvard-led multicenter study found a significant decrease in asthma exacerbations in the first six months of 2020 compared with 2019 (–0.47 exacerbations per year [95% confidence interval (CI), –0.76 to –0.19; *P* = 0.001], a relative reduction of 41%) (9).

In a large UK National Health Service Trust hospital, a significant reduction in all-cause and exacerbation-related asthma and COPD admissions (∼30% and 40%, respectively) was observed, although patients also reported a subjective decline in disease control and a negative impact on their mental health (10). Also in the United Kingdom, a study of a primary care database of 9,949,387 patients containing 100,165 patients with asthma found a significant reduction in attendance to primary care for asthma exacerbations during the pandemic in all age groups, both sexes, and across most regions in England (11).

During pandemic measures, a Japanese survey of 10,226 inpatient subjects diagnosed with asthma exacerbations in 83 hospitals between October 2018 and September 2020 found a >70% decrease in pediatric patients with asthma exacerbations requiring hospital admission (12).

In Guangzhou, China, strict countermeasures undertaken for the pandemic were associated with a decreased frequency of infectious respiratory diseases and severe asthma exacerbations among urban children (13). The authors speculated that this may be because of reduced pollution as well as a reduction in the transmission of viral respiratory infections (13). An increase in the frequency of mild asthma exacerbations was attributed to overlap of symptoms associated with coronavirus disease (COVID-19) and a general fear of development of COVID-19 (13).

In Singapore, a sustained reduction in asthma admissions with PCR-proven respiratory viral infections coincided with the widespread adoption of public health measures (14). The total number of asthma admissions per month dropped from a mean of 64.7 (SD ± 9.1) before the pandemic to 39.2 (±7.5) during the pandemic (*P* < 0.001). During the pandemic, only 11.5% (33 of 288) of asthma admissions had a concurrent PCR-proven respiratory viral infection, whereas one-half (53.5%, 348 of 651) of asthma admissions had a positive result before the pandemic (odds ratio, 0.11; 95% CI, 0.08–0.17; *P* < 0.001). Notably, over a 5-month period from May to September 2020 and onwards, zero asthma admissions had concomitant respiratory viral infections.

A study from Jordan of 1,207 pediatric asthma exacerbations found that with nonpharmacological interventions in place, there was a decrease in exacerbations measured by admissions and emergency room visits (15). During the lockdown (March 22 to May 1, 2020), the mean weekly admissions (2.6 ± 1.4) were significantly lower than those before the lockdown (8.6 ± 2.0) and after the lockdown (5.2 ± 2.0) and significantly lower than in the same weeks in 2019 and 2018 (15).

In Holland, a study of 67 patients with severe and uncontrolled asthma enrolled in a clinical trial (the BREATHE [Better Respiratory Education and Treatment Help Empower] study) showed a significantly reduced (∼70%) asthma exacerbation frequency during COVID-19 social distancing measures compared with that during previous years (16). Anxiety toward acquiring COVID-19 infection was increased in these subjects (16).

### COPD

Equally impressive decreases in exacerbation rates have been reported internationally for patients with COPD. Within the United Kingdom, an interrupted time series analysis of the entire populations of Scotland and Wales (approximately 5.5 and 3.2 million people, respectively) showed a 48% pooled reduction in acute exacerbations of COPD requiring hospital admission (17). Within Wales, emergency room attendance for exacerbations was reduced by 46%, and primary care consultations were reduced by 39% below a 5-year average (17). Interestingly, the authors did not find a rebound in events following the release of lockdown but instead a gradual increase in healthcare usage (17).

In the United States, data involving 4,422 COPD admissions to a large multicenter healthcare system in Maryland demonstrated a season-matched 53% decline in COPD admissions during the SARS-CoV-2 pandemic. The demographics and comorbidity profile of those who did attend were similar to those who attended in nonpandemic circumstances. The decline correlated to community viral burden (*r* = 0.73; 95% CI, 0.67–0.78) (18).

The number of exacerbations of COPD in Hong Kong fell by 44% in the first 3 months of 2020 compared with the same interval in 4 previous years, which was attributed to increased masking and social distancing (19). In Malta a 54.2% drop in acute exacerbation COPD admissions was seen in 2020 (*n* = 119 vs. *n* = 259 in 2019). There were no significant differences in patient demographics or medical comorbidities (20).

Studies from the Singapore General Hospital showed that acute COPD admissions per month decreased by more than 50% (average, 36; SD, 6) during February–July 2020 compared with 92 (SD, 18) before the pandemic (21). Within admitted patients, the rate of positive respiratory viral PCR tests fell from 30% to 10.6%, despite increased PCR testing from 60% of patients before the pandemic to 98% (21).

### Cystic Fibrosis, Bronchiectasis, and Interstitial Lung Disease

Exacerbations also cause progressive declines in lung function in patients with cystic fibrosis (CF) and bronchiectasis. A comparison of exacerbation rates at the CF Centre in Indianapolis, Indiana, in the first months of 2019 (before the pandemic) and 2020 reported a 50% fall that was attributed to restrictions on social interaction and reduced exposure to respiratory viral infections (22). In a prospective UK study of bronchiectasis, the proportion of patients experiencing a hospitalization because of severe exacerbation was 8.8% between March 2020 and March 2021 compared with 14.3% and 16.3% in the 2 previous years (23).

Interstitial lung disease (ILD) is another chronic respiratory disease with poorly understood episodes of exacerbation. A questionnaire survey of 134 hospitals in Japan of acute exacerbations of ILD early in the COVID-19 epidemic found no clear trends in exacerbation frequencies (24). This mitigates against an infective element in ILD exacerbations.

### Confounding Factors

Factors other than infections may have added to the decline in attendances of exacerbations of airway disease. The recognition that COVID-19 infection is associated with worse outcomes in patients with asthma (25) and patients with COPD (26) may have affected patient behavior, and data from the United Kingdom suggest that the reduction in asthma exacerbations may be related to reductions in primary care contacts (27). However, another UK study reported that the fall in primary care attendance for exacerbations for asthma was not seen in attendance numbers to the emergency room, implying that people were struggling to access primary care, or that they were preferentially attending the emergency room or waiting at home until they became severe enough to attend a hospital (11). Others have suggested that the SARS-CoV-2 pandemic may have been an opportunity for patients to take more control over their health care, becoming more adherent to their medications and shielding advice (28).

Air pollution is another factor for consideration. Lockdown events reduced the population-weighted concentration of nitrogen dioxide and particulate matter concentrations by about 60% and 31% in 34 countries, with mixed effects on ozone (29), possibly affecting asthma and COPD exacerbation rates.

Nevertheless, although marked decreases have been reported in admissions for disorders of the respiratory system in the United Kingdom, no changes in admissions for surgery or accidental injury have been observed (30). It is difficult to discount that the declines in exacerbation rates are remarkably consistent internationally and are from comprehensive studies across a wide range of different healthcare systems and environments.

## Acute Bacterial Infections

Lower respiratory bacterial infections are leading causes of global morbidity and mortality, especially in children and older adults (31). During 2016, *Streptococcus pneumoniae* was estimated to have caused approximately 1.1 million deaths worldwide, with *Haemophilus influenzae* also of global importance (31). In common with *Neisseria meningitidis*, which causes meningitis and sepsis, these World Health Organization priority pathogens are transmitted by the respiratory route and are commonly carried in the oropharynx of healthy individuals.

The international Invasive Respiratory Infection Surveillance initiative prospectively analyzed the incidence of invasive disease due to *S. pneumoniae*, *H. influenzae*, and *N. meningitidis* from laboratories in 26 countries and territories across six continents (32). Numbers of weekly cases in 2020 were compared with corresponding data for 2018 and 2019. All countries and territories had experienced a significant and sustained reduction in invasive diseases due to *S. pneumoniae, H. influenzae*, and *N. meningitidis* in early 2020 (January 1 to May 31, 2020), coinciding with the introduction of COVID-19 containment measures in each country.

Overall, social changes caused by the SARS2-Cov-19 pandemic were accompanied by a 38% decrease in the incidence of reported *S. pneumoniae* invasive infections (incidence rate ratio [IRR], 0.62 [95% CI, 0.54–0.70]). Similar steep decreases were seen for *H. influenzae* and *N. meningitidis* infections (32). The authors estimated population mobility changes from mobile phone data, and using time series analysis showed a decrease in reported *S. pneumoniae* infections of 68% at 4 weeks (IRR, 0.32 [95% CI, 0.27–0.37]) and 82% at 8 weeks (0.18 [0.14–0.23]) after the week when movement changes were first observed (32). By contrast, the incidence of disease due to *Streptococcus agalactiae*, a nonrespiratory pathogen, did not change during the pandemic.

In the UK Prospective National Cohort Study, the incidence of invasive pneumococcal disease in all of England fell by 30% in the study year between 2019 and 2020 compared with 2018 to 2019 (IRR, 0.70; 95% CI, 0.18–2.67), with large reductions observed across all age groups during March–June 2,020 (33). Week-by-week contrasts during the “Circuit Breaker” partial lockdown in Singapore against the preceding 10 years showed the mean number of positive urinary streptococcal antigen tests in 2020 to have fallen to by 50% compared with 2010–2019 (34).

In Taiwan, invasive pneumococcal disease (IPD) is a notifiable condition for which reporting is mandatory. A comparison of the case number of patients with IPD from Taiwan's Centers for Disease Control between January and August found 162 IPD cases were reported during the first 8 months in 2020, compared with a monthly range of 282 to 400 cases over the previous 4 years (35). In Guangzhou, China, “strict childhood pneumonia” cases fell from more than 600 over each of the past 3 years to 172 in 2020 (13). In Holland, a study of three hospitals found that the first COVID-19 wave in March, April, and May 2020 was marked by 13 adults hospitalized with pneumococcal bacteraemia, compared with 32 ± 6 cases during the corresponding months in the preceding 5 years (36).

## Postpandemic Return of Common Viral Illnesses

The relaxation of the most stringent public health interventions in many countries has been followed by a rapid resurgence in rates of seasonal respiratory viral infections. For example, the Centers for Disease Control and Prevention have reported that reduced transmission of common respiratory viruses in the United States during 2020 was followed by increased RSV activity from April 2021, and increased rates of infection with coronaviruses, parainfluenza viruses, and respiratory adenoviruses from January or February 2021. By contrast, HRV and enteroviruses began to increase in June 2020 (37). An early resurgence of HRV has also been observed in German national data (38).

Data from approximately 260 hospitals and clinics in Tokyo have numbered pediatrician-diagnosed weekly cases of RSV infection since 2003 (39). No outbreaks of RSV were reported in 2020, but in 2021, the largest annual increase in cases since monitoring began was observed. Following relaxation of physical distancing recommendations in Australia, RSV activity increased well beyond median yearly peaks in 2021 (40). Both in Japan and in Australia, the median age of patients with RSV was significantly higher during resurgence than in previous years (39, 40), suggesting that an accumulation of susceptible persons during the pandemic may have contributed to this subsequent large outbreak.

The unusual timing and magnitude of the resurgent viral infections raise complex clinically relevant questions about the contribution of birth cohort effects, natural immunity, and interventions (37).

## Known Roles of Viral Exacerbations in Common Respiratory Diseases

Exacerbations of childhood asthma have long been recognized to be precipitated by infections with common respiratory viruses, among which HRV is by far the most important pathogen (41, 42). Adult asthma exacerbations show a similarly close relationship to HRV infection (43).

COPD exacerbations too are triggered by viral infections (44, 45). For example, in a longitudinal UK study, 40% of COPD exacerbations were associated with viral infections (46), and HRV was found in 58% of viral exacerbations. Other viruses included coronavirus (11% of virus exacerbations), influenza A and B (16%), and occasional parainfluenza and adenovirus detections. RSV was detected in approximately 29% of exacerbations, although RSV was also found in a significant number of patients in the stable state (46). Exacerbations were more severe objectively and symptomatically when viruses were present (46).

In normal circumstances, different viruses circulate in populations at different times, and this is reflected in the age of patients and the nature of their exacerbations. In the Northern hemisphere, childhood asthma exacerbations peak following school return after the summer vacation and are predominantly associated with HRV (47). In older subjects, exacerbations of both COPD and adult asthma, with increasing risk with age, are at their highest average annual prevalence during Christmas. This appears to be independent of the timing of prevalence of influenza, RSV, parainfluenza, or adenovirus detections (47). The role of HRV during the winter peak of both diseases has not been clarified, and transmission of bacterial pathogens to patients with COPD (discussed below) is also a factor.

In patients with CF, the frequency of viral respiratory infections also closely associates with pulmonary deterioration (48). In children with CF, 46% of exacerbations have been associated with respiratory viruses, compared with asymptomatic carriage in 17% (49). Viral infections are recognized in 33% of adult CF exacerbations (50) and are most commonly because of HRV (51). It has been suggested that respiratory viruses may represent an underexploited target in the battle to control CF symptoms and progression (52). Respiratory viruses, most frequently HRV-A, are similarly commonly detected during pulmonary exacerbations of bronchiectasis in children (53).

## Bacterial Transmission and Interactions with Viruses

Pathobionts are normally resident bacteria that in some circumstances can cause disease (54). *S. pneumoniae,* nontypeable *H. influenzae* (NTHi), *N. meningitidis*, and *Moraxella catarrhalis* are classical pathobionts that are commonly found in normal airways. Transmission of *S. pneumoniae,* NTHi, and *N. meningitidis* from healthy carriers is important in invasive bacterial diseases (55–57).

Recurrent exacerbations of COPD in individual patients are associated with the isolation of new strains of *S. pneumoniae,* NTHi, and *M. catarrhalis* (58), supporting the causative role of bacteria and in the current context suggesting that their transmission may be suppressed to therapeutic advantage.

There is also strong evidence for bacterial pathogen engagement in asthmatic airway inflammation. Bisgaard and colleagues found by culture that neonatal nasopharyngeal colonization with *S. pneumoniae*, *M. catarrhalis*, or *H. influenzae* foreshadowed the development of asthma (59). We subsequently discovered by bacterial sequencing that similar organisms were in excess in the lower airway microbiota of children and adults with asthma (60). *Proteobacteria* excess has now consistently been found in asthmatic airways (60–62) (reviewed in Reference 63), as have *Streptococcus* spp. in severe disease (60, 64, 65). The neonatal study of Bisgaard and colleagues (59) and the presence of significant differences in wheezing-associated pathobiont frequencies in children who are naive to the use of antibiotics and inhaled steroids (66) indicate that these changes are not secondary to asthma therapy.

Viral perturbations of the resident microbiome may be a general initiating factor of severe bacterial infections (67). Interactions between respiratory tract viruses and resident pathobionts are well recognized in upper respiratory tract infections (68). *H. influenzae* is the most common bacterial accompaniment of COPD exacerbations (69), and its presence during exacerbations with HRV is associated with poor outcomes (70). Similarly, the presence of pathogenic bacteria during HRV infection is associated with asthma exacerbations (71). Potential mechanisms for interactions are reviewed in Reference 72.

Most deaths in the 1918–1919 influenza pandemic were attributable to secondary pneumonia caused by *S. pneumoniae* and *H. influenzae* (73), when the mass movement of troops and people contributed to bacterial as well as viral propagation. The recent pandemic-associated reduction in global rates of pneumonia (described above) was thought by the Invasive Respiratory Infection Surveillance authors to follow reduced transmission of pathogenic bacteria (31), although they also recognized that respiratory viruses have a role in bacterial disease (5, 74).

Of interest in this regard is a prospective study from Israel of pneumococcal pneumonia in young children (75). The authors observed a steep decline in the incidence of community-acquired alveolar pneumonia and bacteraemic pneumococcal pneumonia during the pandemic (IRRs of 0.07 and 0.19, respectively). However, the prevalence of pneumococcal carriage was only slightly reduced, and the density of colonization and pneumococcal serotype distributions were similar to those in previous years. At the same time, the pneumococcus-associated disease decline was associated with a suppression of RSV, influenza viruses, and human metapneumovirus, often implicated as copathogens with pneumococcus (75).

## Ecology of Airway Microbial Communities

**Figure 1.**
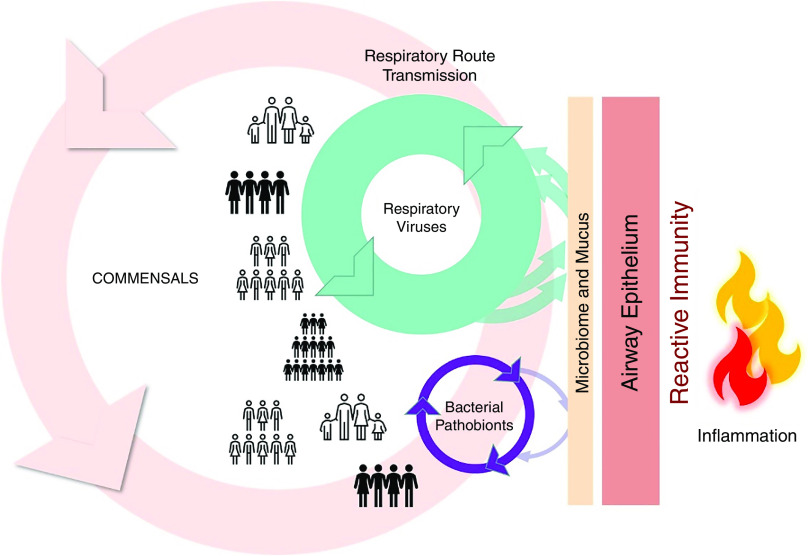
Major microbial factors in acute respiratory episodes. To the left of the figure, the circulation of multiple respiratory viruses in the population provides a continued source of mucosal insults. Circulation and adherence of common bacterial pathogens has the potential to cause invasive disease and sepsis as well as lower-grade chronic damage. Airway commensals also circulate within the population, and their diversity is protective against infection. Viruses and bacteria interact positively and negatively within the mucous layer and the epithelium. The microbiota and epithelia induce reactive immunity to infection and consequent inflammation, shown on the right. Current therapies and research investment are directed to the right, but consequences of the severe acute respiratory syndrome coronavirus 2 (SARS-CoV-2) pandemic show the extraordinary potential of left-sided interventions.

The marked effects of social isolation during the SARS-CoV-2 pandemic encourage an overview of interactions between the population and airway pathogens (Figure 1). Respiratory viruses and bacterial pathobionts are in general circulation within the community and are transmitted over relatively short time scales between healthy and susceptible individuals (Figure 1, left side). Commensal microbial communities at the airway mucosal barrier are conserved and highly ordered (76), reflecting symbiosis and coevolution with human host factors (77). They play essential roles in resistance to pulmonary infections (62, 78, 79). Over longer periods (possibly generations), loss of commensal diversity in the wider population may reduce pathogen resistance (80). The clinical emphasis in asthma and in COPD has understandably been directed against inflammation (Figure 1, right side), but the likely efficacy of left-sided interventions to prevent exacerbations is now clear.

## Therapeutic Implications

Modern biologic therapies in controlled clinical trials have successfully reduced exacerbations rates of moderate to severe asthma (81). By analogy, glucocorticoids and biologics are beneficial in the treatment of the inflammatory consequences of SARS-CoV-2 infection (82, 83). Nevertheless, nearly two-thirds of patients with severe asthma treated with biologics continue to experience uncontrolled disease (84). Although the importance of virus infections at the start of acute asthma exacerbations is very well understood, it may be fair to say that their prevention and treatment has before now been neglected. Indeed, a recent influential publication failed to mention infection at all in a review of potential of strategies to drive down the global burden of asthma (85).

The role of bacterial infections is accepted in patients with COPD, but viruses are not usually treated. Notwithstanding the known contribution of viruses, patients with recurrent COPD exacerbations typically are managed with repeated systemic antibiotic courses (86). However, microbiological diagnosis by culture of NHTi is demanding (79), and antibiotics are often given empirically. Acute infection accompanying COPD is one of the most common indications for adult antimicrobial therapy and plays a substantial role in antimicrobial resistance in the population (87). Consequently, sequence-based distinction of viral and bacterial components may better target management.

**Figure 2.**
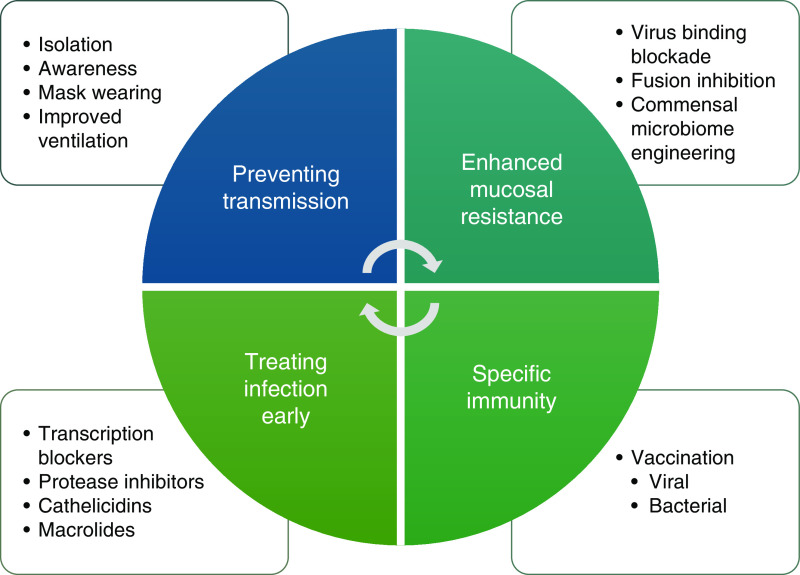
Prevention and treatment of viral-induced exacerbations. The figure illustrates potential ways of mitigating the effects of respiratory viral infections on exacerbations of chronic obstructive pulmonary disease and asthma. Nonpharmaceutical interventions have been of proven efficacy in preventing transmission but may come at a significant societal cost. Novel methods to block viral adhesion and invasion of the mucosa have a high potential. Vaccination can provide effective immunity against severe infections, although it has so far proved difficult for important viral and bacterial pathogens. A range of drugs are already available for treating active viral infections, but strategies have yet to evolve for their early use.

The efficacy of innovative responses to the SARS-CoV-2 pandemic demonstrates that several levels of strategy directed against microbial infection (Figure 2) are not only possible but also likely to be successful. For those approaches already developed, this review strengthens the case for their clinical implementation, but the opportunity is also clear for novel interventions. We consider preventive and therapeutic approaches below. We consider that most preventive therapies could be administered at times of high risk, such as autumn for childhood asthma in the winter months for patients with adult asthma and COPD.

### Nonpharmaceutical Interventions

The unexpected decreases in exacerbations of chronic respiratory diseases have resulted from a diverse array of different public health measures applied in different countries. Some measures such as social isolation and school lockdowns are not pragmatic on a long-term basis, and it remains unclear which interventions should be pursued in the postpandemic era. Prospective research is therefore urgently required to assess the impact of individual interventions and should include objective measurements of viral transmission and kinetics in susceptible populations. In addition, the importance of public education should be highlighted regarding the risk that apparently trivial colds may pose to vulnerable individuals.

Access to safe air through sophisticated ventilation has potential in schools and workplaces (88), but a challenge exists to improve ventilation in the developing world. Hospitals are high-risk environments for patients and for staff. As a precedent, the transmission of respiratory bacterial pathogens between susceptible individuals is well recognized in hospitals (89) and in children attending clinics for cystic fibrosis (90–92). *Mycobacterium abscessus* is an exemplar for the international spread of dominant clones of an important lung pathogen (93). Knowledge of these risks to patients with CF has led to aggressive measures to control transmission of multiple drug resistant strains in hospitals and clinics (94). Nosocomial infections by HRV and common bacterial pathogens may also respond to such measures.

### Bolstering Mucosal Resistance

The healthy airway microbiota are contained within a structured ecosystem, suggesting balanced relationships between the microbiome and human host factors (76). Although still poorly understood, this airway microbiome-mucosal complex is likely to exhibit cognate effects on pathogen activity and reactive immunity and is a rich area for future study and manipulation.

Airway microbiome-mucosal complex activities may be enhanced by nonspecific “trained immunity” to a range of viral infections. Bacille Calmette-Guérin vaccination in children protects against a range of serious infections independently of tuberculosis prevention (95) and in elderly patients doubles the time to occurrence of respiratory tract infections of probable viral origin (96). Other nonspecific approaches might include an oral bacterial extract (97), currently being investigated in a controlled clinical trial (the ORBEX [Oral Bacterial Extract] study: NCT02148796) for the prevention of wheezing lower respiratory tract illness.

### Binding Inhibition

HRV gains access to airway epithelial cells by binding to surface receptors. Major group HRVs bind to ICAM-1 (intercellular adhesion molecule 1) (98), and minor group viruses bind the LDLR (low-density lipoprotein receptor) (99). HRV-C, which is associated with severe acute asthma attacks more frequently than other rhinoviruses (reviewed in Reference 100), binds to CDHR3 (cadherin-3) (101). This limited range of receptors may permit strategies such as competitive inhibition (102) to prevent virus binding to airway epithelial cells. The initial site of infection is often nasal, providing the opportunity for topical therapies.

Most viral pathogens are membrane-enveloped viruses that require the fusion of viral and cell membranes for virus entry. Compounds that target the membrane fusion process represent new possibilities for broad-spectrum antiviral discovery (103).

It may be relevant that the most important genetic effect on asthma (the *ORMDL3* and *GSDMB* locus) (104) strongly mediates the risk of viral induced exacerbations (105) and provides potentially druggable targets in sphingolipid pathways that may influence HRV adhesion by modulating expression of ICAM-1 (106). *CDHR3* is another susceptibility locus for early childhood asthma with severe exacerbations (107). The asthma-associated coding polymorphism (*CDHR3 C529Y*) exhibits enhanced cell-surface expression of protein and has shown 10-fold increases in HRV-C binding and virus progeny yields in a cellular model (108).

### Vaccines

Vaccines against bacterial respiratory pathobionts can be highly effective and are administered internationally. The vaccine-related loss of capsular genes in NTHi and the widening number of circulating strains has, however, led to an urgent and ongoing search for alternative antigens (87).

Prevention of HRV infections by vaccination has also been difficult to achieve. HRV is made up of three genetically distinct groups, designated A, B, and C and containing more than 100 serotypes (109). Multiple virus types circulate simultaneously in families, and HRV are frequently transmitted from children to other family members (110). HRV sequences show minimal common sites that might be antigen epitopes, so single vaccines have been problematic to design (111). Polyvalent vaccines may be useful (112), although development of a polyvalent vaccine for Dengue, an infection caused by four flaviviral serotypes (DENV1–4), has been hindered by antibody-dependent enhancement of disease after mixed secondary infections (113).

Despite these difficulties, the technological advances underpinning rapid development of vaccines for SARS-CoV-2 (114) should offer great hope for future efforts.

### Small Molecule

HRV infections are an obvious target for drug therapy, although major challenges have been recognized (115). Approaches used include ribavirin, capsid binding inhibitors, 3C protease inhibitors, NO enhancers, and mammalian cathelicidins LL-37, protegrin-1, and SMAP-29 (reviewed in Reference 115). Molnupiravir, a novel antiviral recently identified as efficacious against SARS-CoV-2 (116), is a prodrug for the ribonucleoside analog β-D-N4-hydroxycytidine, which has broad-spectrum antiviral activity against RNA viruses, including influenza (117). The macrolide antibiotic azithromycin is effective in preventing exacerbations of COPD (118), and it is of interest that azithromycin reduces *in vitro* replication of several classes of viruses, including rhinovirus, influenza A, and coronaviruses, via mechanisms that include enhanced expression of antiviral pattern-recognition receptors and induction of antiviral type I and III IFN responses (119). These experiences and the potential size of the market encourage industry efforts to bring to the clinic small molecules to treat HRV.

## Conclusions

Viral infections have long been recognized to precipitate attacks of asthma and COPD, but little of this knowledge has translated to improvements in healthcare. Bacterial pathobiont transmission also plays a significant but underestimated part. The SARS-CoV-2 pandemic demonstrates how targeting of common respiratory pathogens could prevent 50% of exacerbations of COPD and asthma. The successful scientific response to SARS-CoV-2 should encourage a reappraisal of means to prevent or mitigate other universal respiratory infections. The efficacy of pooling of resources during the pandemic into large, multiarm, multicenter, multicountry randomized clinical trials (120) suggests that similar efforts are justified for common respiratory pathogens.
